# 

**Published:** 1857-06

**Authors:** 


### CONTENTS OF HALL'S JOURNAL OF HEALTH,

FROM JANUARY TO JUNE, 1857.

Antecedents, 87.
Architecture, Defective, 107.

Beard Wearing, 71.
Benevolent Men, 126.
Bodily Carriage, 8.
Bodily Endurance, 90.
Burying Alive Prevented, 35.

Children's Reading, 44, 142.
Christian Missions, 91.
Church and Theatre, 113.
Civility, 97.
Clergymen's Children, 129.
Clerical Health, 11.
Coffee, 147.
Common Sense, 19.
Constitutions Ruined, 117.
Consumption, 77, 49.
Cornaro and Cornelius, 66, 144.

Death Made Easy, 143.
Disease and Crime, 5.
Divining Rods, 139.
Dr. E. Nott's Munificence, 65.
Dr. Livingston, 92.
Drinking at Meals, 115.

Eating and Drinking, 63.
Eating, A Year's, 33.

Family Music, 21.
Flour too White, 69, 102.

Getting Worse, 120.
Gloves and Shoes, Tight, 36.

Healthy Houses, 106.
Heart Diseases, 81.
Horseback Exercise, 80.
How toGrow Beautiful, 57.
How to Live Long, 66.

Insanity, 110.
Insanity and Suicide, 16.
Insanity of Blacks, 21.
Insect Bites Cured, 43, 51.
Inverted Toe Nails, 97.
Is it Right? 122.
Isms of the Times, 111.

Journal's Work, 46, 58.

Kentucky's Daughters, 103

Life's Last Sight, 47.
Living 100 Years, 66.

Make Home Happy, 41.
Milk, Use of, 64.
Mistress' Rules, 137.
Morning Walks, 30.
Mother, Watchful, 142.

Occupation on Health, 49.

Out-Door Activities, 80.

Painless Cure of Corns, 32.
Plant a Vine, 94.
Pone, 101.
Position in Sleeping, 45.

Railroad Precautions, 14, 48.
Rat Exterminator, 51.

Scarlet Fever, 53.
Secret Remedies, 122
Servant Plague, 132.
Servants' Rules, 134.
Snake-Bite Cured, 51.
Soap Suds, 121.
Spinal Affections, 81.
Spiritualism, 37, 139.
Supporters, Bodily, 35.

Tea and Coffee, 63.
Theatre, its Effects, 129.
The Early Dead, 51.
Thomas H. Benton, 146.
Thoughts for Parents, 50.
Throat Ail Causes, 145.
To be Safe, 145.
To Make Corn Bread, 105
Tooth-Wash, Best, 32.
Travelling for Health, 69.

Waste and Want, 133.
Water Cure, 89.

